# Serum-based BRAF V600E detection improves early diagnosis of papillary thyroid carcinoma: A prospective diagnostic accuracy study compared with ultrasound-guided cytology

**DOI:** 10.1097/MD.0000000000049200

**Published:** 2026-06-12

**Authors:** Qi Li, Yiyan Lu, Xiangli Li, Jing Jiang, Xiuqing Wang, Jia Xu, Qi Zhang, Longzhu Zhao

**Affiliations:** a Department of Pathology, Beijing Integrated Traditional Chinese and Western Medicine Hospital, Beijing, China; b Department of Pathology, Aerospace Center Hospital, Beijing, China; c Department of Pathology, The First Affiliated Hospital of Tsinghua University, Beijing, China; d Department of Ultrasound, Beijing Integrated Traditional Chinese and Western Medicine Hospital, Beijing, China; e Department of Pharmacy, Beijing Integrated Traditional Chinese and Western Medicine Hospital, Beijing, China; f Department of Rehabilitation, Beijing Integrated Traditional Chinese and Western Medicine Hospital, Beijing, China; g Department of Otolaryngology-Head and Neck Surgery, Beijing Integrated Traditional Chinese and Western Medicine Hospital, Beijing, China.

**Keywords:** BRAF V600E, diagnostic accuracy, papillary thyroid carcinoma, serum biomarkers, ultrasound-guided fine-needle aspiration cytology

## Abstract

Accurate preoperative diagnosis of papillary thyroid carcinoma (PTC) remains difficult, especially for small thyroid nodules and lesions with indeterminate cytology. This prospective diagnostic study evaluated the clinical value of serum-based v-Raf murine sarcoma viral oncogene homolog B1 (BRAF) V600E mutation detection and its performance in combination with ultrasound-guided fine-needle aspiration cytology (US-FNAC). A total of 145 patients with thyroid nodules classified as TI-RADS 3 to 5 and measuring 3 cm or less were enrolled. All patients underwent US-FNAC and serum BRAF V600E testing using allele-specific quantitative PCR, with postoperative histopathology used as the reference standard. Among the enrolled patients, 64 were diagnosed with PTC and 81 had benign nodules. US-FNAC showed a sensitivity of 68.75% and specificity of 83.95%, while serum BRAF V600E testing showed a sensitivity of 62.50%, specificity of 81.48%, and an area under the curve (AUC) of 0.792. In patients with papillary thyroid microcarcinoma, serum BRAF V600E testing demonstrated higher sensitivity than US-FNAC, 94.12% versus 64.71%, with an AUC of 0.841. However, its diagnostic sensitivity remained limited in poorly differentiated PTC, at 57.14%. A combined model integrating US-FNAC and serum BRAF V600E testing improved diagnostic performance, with a sensitivity of 81.25%, specificity of 85.19%, and AUC of 0.873, significantly outperforming either method alone. These findings suggest that serum BRAF V600E detection is a useful complementary, minimally invasive approach for diagnosing PTC, particularly micro-PTC, and may improve early detection and risk stratification when combined with US-FNAC.

## 1. Introduction

Papillary thyroid carcinoma (PTC) is the most common histological subtype of thyroid malignancy and accounts for more than 90% of all thyroid cancer cases worldwide.^[1]^ Although the overall prognosis of PTC is favorable, with a 10-year survival rate exceeding 95%, its rapidly increasing incidence over recent decades has introduced significant challenges in early and accurate diagnosis.^[1,2]^ In particular, the widespread use of high-resolution imaging has led to increased detection of small thyroid nodules and papillary thyroid microcarcinomas (micro-PTC, ≤1 cm), where distinguishing clinically significant malignancies from indolent or benign lesions remains difficult.^[3]^ This diagnostic uncertainty contributes to both underdiagnosis of aggressive disease and overtreatment of low-risk nodules, ultimately increasing healthcare burden and patient morbidity.^[2–4]^

Ultrasound-guided fine-needle aspiration cytology (US-FNAC) is currently the cornerstone of preoperative evaluation for thyroid nodules, with reported diagnostic accuracy ranging from 85 to 95%.^[3–5]^ However, its performance is influenced by sampling adequacy, operator expertise, and intrinsic cytological characteristics of the lesion. Notably, approximately 10 to 30% of nodules are classified as indeterminate (Bethesda III/IV), posing a persistent clinical dilemma in decision-making.^[5,6]^ These limitations are further amplified in small nodules, cystic lesions, or tumors with subtle cytological features, where insufficient cellularity or atypia may reduce diagnostic reliability.^[7,8]^ Therefore, there is an urgent need for complementary diagnostic approaches that can enhance risk stratification while minimizing reliance on invasive procedures.

Molecular diagnostics has emerged as a promising strategy to address these limitations. Among the known genetic alterations in thyroid cancer, the v-Raf murine sarcoma viral oncogene homolog B1 (BRAF) V600E mutation is one of the most extensively studied and clinically relevant biomarkers in PTC, with a reported prevalence of 40 to 80% in classical cases.^[7–10]^ As an early oncogenic driver within the mitogen-activated protein kinase signaling pathway, BRAF V600E is closely associated with tumor initiation, progression, and aggressive clinicopathological features.^[11–14]^ Consequently, BRAF mutation testing has been increasingly incorporated into diagnostic workflows, particularly in cytologically indeterminate nodules, where it can improve diagnostic confidence.^[9,10]^ However, most existing studies have focused on mutation signals in tissue or fine-needle aspiration samples, while the clinical utility of noninvasive serum-based BRAF V600E detection remains insufficiently investigated.

In parallel, the molecular landscape of thyroid cancer diagnostics continues to evolve, with additional biomarkers such as TERT promoter mutations, RAS gene alterations, and microRNA expression profiles demonstrating potential value in improving diagnostic and prognostic stratification.^[6]^ Despite these advances, many of these approaches are limited by higher costs, technical complexity, or dependence on tissue-based sampling. In contrast, a serum-based assay targeting a well-established driver mutation may offer a more practical, minimally invasive alternative if adequate diagnostic performance can be demonstrated. Importantly, such an approach may be particularly beneficial in clinically challenging scenarios, including micro-PTC, indeterminate nodules, and poorly differentiated tumors, where conventional cytology may be insufficient.

Therefore, the present prospective study was designed to evaluate the diagnostic efficacy of serum BRAF V600E mutation signal for early PTC diagnosis and to directly compare its performance with US-FNAC using postoperative histopathology as the reference standard. In addition to overall diagnostic accuracy, we specifically assessed its performance across clinically relevant subgroups, including micro-PTC and poorly differentiated PTC. By clarifying the complementary role of a noninvasive molecular assay within the current diagnostic pathway, this study aims to contribute to the development of a more precise and clinically applicable strategy for early thyroid cancer detection.

## 2. Materials and methods

### 2.1. Study design and participants

This prospective diagnostic accuracy study was conducted at the Department of Thyroid Surgery, Beijing Integrated Traditional Chinese and Western Medicine Hospital, between April 2023 and August 2025. The study protocol was reviewed and approved by the Ethics Committee of Beijing Integrated Traditional Chinese and Western Medicine Hospital (Approval No.: BITC2025). All procedures were conducted in accordance with the principles of the Declaration of Helsinki. Written informed consent was obtained from all participants prior to enrollment.

Sample size estimation was performed using G*Power 3.1 based on anticipated sensitivities of 75 to 80% for US-FNAC and 80 to 85% for BRAF V600E detection, with a two-sided α of 0.05, statistical power (1−β) of 0.80, and an assumed inter-test correlation coefficient (ρ) of 0.5. Accounting for a 10% dropout rate, the minimum required sample size was calculated to be 145 patients.

Eligible participants were adults aged 18 to 75 years presenting with thyroid nodules (maximum diameter ≤ 3 cm) classified as Thyroid Imaging Reporting and Data System (TI-RADS) categories 3 to 5 on ultrasonography. All patients were scheduled to undergo ultrasound-guided fine-needle aspiration cytology (US-FNAC) and/or surgical intervention. Exclusion criteria included: nodules > 3 cm or presence of clinically significant cervical lymphadenopathy (short-axis diameter > 8 mm); prior thyroid surgery or biopsy within one month; contraindications to fine-needle aspiration cytology (FNAC) (INR > 1.5 or platelet count < 100 × 10^9^/L); pregnancy or lactation; and incomplete pathological data.

A total of 145 patients were included in the final analysis. Postoperative histopathology served as the reference standard, confirming 64 cases of PTC and 81 benign thyroid nodules. Baseline demographic and clinical characteristics are summarized in Table 1.

**Table 1:** Baseline characteristics of the study population (n = 145).

Variable	Value
Age (yr)	63.40 ± 5.81
Sex, n (%)	
Male	24 (16.6)
Female	121 (83.4)
BMI (kg/m^2^)	22.41 ± 2.09
TI-RADS category, n (%)	
3	42 (29.0)
4	77 (53.1)
5	26 (17.9)
Smoking status, n (%)	
Yes	94 (64.8)
No	51 (35.2)
Alcohol consumption, n (%)	
Yes	46 (31.7)
No	99 (68.3)

TI-RADS = Thyroid Imaging Reporting and Data System.

To minimize bias, cytological evaluation and molecular testing were performed independently by blinded investigators who were unaware of each other’s results and final histopathological outcomes.

This study was designed and reported in accordance with the Standards for Reporting Diagnostic Accuracy Studies (STARD) 2015 guidelines for diagnostic accuracy studies.

### 2.2. Ultrasound-guided fine-needle aspiration cytology (US-FNAC)

US-FNAC was performed using a standardized protocol by 2 experienced ultrasound physicians (≥5 years of thyroid biopsy experience). Cytological results were classified according to the Bethesda System for Reporting Thyroid Cytopathology. For diagnostic performance analysis, Bethesda categories V–VI were considered positive for malignancy, while category II was considered benign. Indeterminate categories (III/IV) were analyzed separately in subgroup analyses.

### 2.3. Serum DNA extraction and BRAF V600E mutation signal

#### 2.3.1. DNA extraction

Peripheral blood samples were collected preoperatively, and serum was separated by centrifugation. Circulating cell-free DNA (cfDNA) was extracted using the QIAGEN QIAamp DNA FFPE Kit (QIAGEN, Germany) according to the manufacturer’s protocol.

DNA concentration and purity were assessed using Qubit fluorometry (target ≥ 20 ng/μL) and NanoDrop spectrophotometry (OD260/280 ratio: 1.8–2.0). DNA integrity was evaluated by agarose gel electrophoresis.

#### 2.3.2. Quantitative detection of BRAF V600E mutation

BRAF V600E mutation analysis was performed using allele-specific quantitative real-time PCR (qPCR) with TaqMan probes. The reaction mixture (20 μL total volume) contained 10 μL of 2 × qPCR premix, mutation-specific primers and probes, reference gene control (ACTB), and 2 μL of template DNA.

Thermal cycling conditions were as follows: initial denaturation at 95°C for 3 minutes, followed by 40 cycles of denaturation at 95°C for 15 seconds and annealing/extension at 60°C for 60 seconds.

Mutation signal was based on amplification curves and cycle threshold (Ct) values using mutation-specific probes. A predefined Ct cutoff value was applied to determine mutation positivity. Importantly, the analysis was based on the mutation signal (qualitative/threshold-based) rather than gene expression quantification, ensuring methodological consistency with mutation-specific assays.

### 2.4. Pre-analytical standardization and quality control

To ensure the reproducibility of serum-based BRAF V600E detection, all pre-analytical procedures were standardized. Peripheral venous blood was collected preoperatively before FNAC or surgical manipulation to minimize contamination from procedure-related cellular release. Blood samples were collected into sterile serum-separation tubes and processed within 2 hours of collection. After clotting at room temperature for 30 minutes, samples were centrifuged at 1600 × g for 10 minutes at 4°C. The serum supernatant was carefully transferred into nuclease-free tubes without disturbing the cellular layer and subjected to a second centrifugation at 16,000 × g for 10 minutes at 4°C to remove residual cells and debris.

Serum aliquots were stored at −80°C until cfDNA extraction. Repeated freeze–thaw cycles were avoided by aliquoting each sample into single-use volumes. Samples showing visible hemolysis, insufficient serum volume, delayed processing, or evidence of contamination were excluded from molecular analysis. cfDNA extraction was performed using the same extraction kit and protocol for all samples, and all assays were conducted under identical reaction conditions. DNA concentration, purity, and integrity were assessed before qPCR analysis. Internal reference gene amplification was used to confirm sample quality, and samples failing predefined quality thresholds were re-extracted or excluded.

This standardized workflow minimized variability related to collection timing, clotting, centrifugation, storage, and DNA quality, thereby improving the reproducibility and reliability of the serum-based BRAF V600E mutation signal.

### 2.5. Outcome measures

The primary outcome was the diagnostic performance of the serum BRAF V600E mutation signal for PTC, using postoperative histopathology as the gold standard.

Secondary outcomes included:

- Diagnostic performance of US-FNAC
- Combined diagnostic performance of BRAF V600E + US-FNAC
- Subgroup analyses in micro-PTC (≤1 cm) and poorly differentiated PTC

### 2.6. Statistical analysis

Statistical analyses were performed using SPSS version 34.0 and MedCalc (or equivalent statistical software for receiver operating characteristic (ROC) comparison).

Continuous variables were expressed as mean ± standard deviation and compared using the independent-samples *t*-test. Categorical variables were presented as counts and percentages and compared using the chi-square test.

Diagnostic performance metrics, including sensitivity, specificity, positive predictive value, negative predictive value, and accuracy, were calculated with 95% confidence intervals (CIs).

ROC curve analysis was used to evaluate diagnostic performance, and the area under the curve (AUC) was calculated. Comparisons between AUCs were performed using the DeLong test.

To evaluate the added value of combined testing, a multivariable logistic regression model incorporating US-FNAC and BRAF V600E status was constructed, and predicted probabilities were used to generate combined ROC curves.

Agreement between diagnostic methods and histopathology was evaluated using Cohen Kappa coefficient.

Potential confounding variables, including age, sex, BMI, TI-RADS category, smoking status, and alcohol consumption, were evaluated in exploratory univariable analyses. Variables not significantly associated with the outcome and not influencing model performance were not included in the final multivariable model to avoid overfitting.

A two-sided *P*-value < .05 was considered statistically significant.

## 3. Results

### 3.1. Diagnostic performance of US-FNAC

Among the 145 patients, 64 were diagnosed with PTC and 81 with benign nodules based on histopathology. US-FNAC identified 44 true-positive and 68 true-negative cases, with 20 false negatives and 13 false positives (Table 2).

**Table 2:** Contingency table of US-FNAC for the diagnosis of papillary thyroid carcinoma.

	PTC (+)	PTC (−)	Total
FNAC (+)	44	13	57
FNAC (−)	20	68	88
Total	64	81	145

FNAC = fine-needle aspiration cytology, PTC = papillary thyroid carcinoma, US-FNAC = ultrasound-guided fine-needle aspiration cytology.

The sensitivity and specificity were 68.75% (95% CI: 56.0–79.7) and 83.95% (95% CI: 74.0–91.2), respectively, with an overall accuracy of 77.24%. The positive predictive value and negative predictive value were 77.19% and 77.27%, respectively (Table 3).

**Table 3:** Diagnostic Performance of US-FNAC and serum BRAF V600E for discrimination of PTC.

Parameter	US-FNAC	BRAF V600E
Sensitivity (%)	68.75 (56.0–79.7)	62.50 (49.5–74.4)
Specificity (%)	83.95 (74.0–91.2)	81.48 (71.2–89.2)
PPV (%)	77.19 (64.5–86.8)	73.91 (62.3–83.1)
NPV (%)	77.27 (66.9–85.5)	71.11 (60.5–80.1)
Accuracy (%)	77.24 (69.5–83.8)	72.41 (64.3–79.5)

Values in parentheses represent 95% confidence intervals. Diagnostic indices were calculated using postoperative histopathology as the reference standard.

BRAF = v-Raf murine sarcoma viral oncogene homolog B1, NPV = negative predictive value, PPV = positive predictive value, PTC = papillary thyroid carcinoma, US-FNAC = ultrasound-guided fine-needle aspiration cytology.

These results indicate moderate sensitivity with relatively high specificity for PTC detection.

### 3.2. Diagnostic performance of serum BRAF V600E for PTC

BRAF V600E mutation signal (Ct-based detection signal) was significantly higher in patients with PTC compared with benign nodules (1.52 ± 0.68 vs 0.89 ± 0.40, *P* < .001; Table 4).

**Table 4:** Comparison of Ct-derived BRAF V600E mutation signal between PTC and benign nodules.

Group	Mean ± SD
PTC	1.52 ± 0.68
Benign	0.89 ± 0.40

Data are presented as mean ± standard deviation (SD). Statistical comparison between groups was performed using an independent samples *t*-test.

BRAF = v-Raf murine sarcoma viral oncogene homolog B1, Ct = cycle threshold, PTC = papillary thyroid carcinoma.

**Figure 1.**
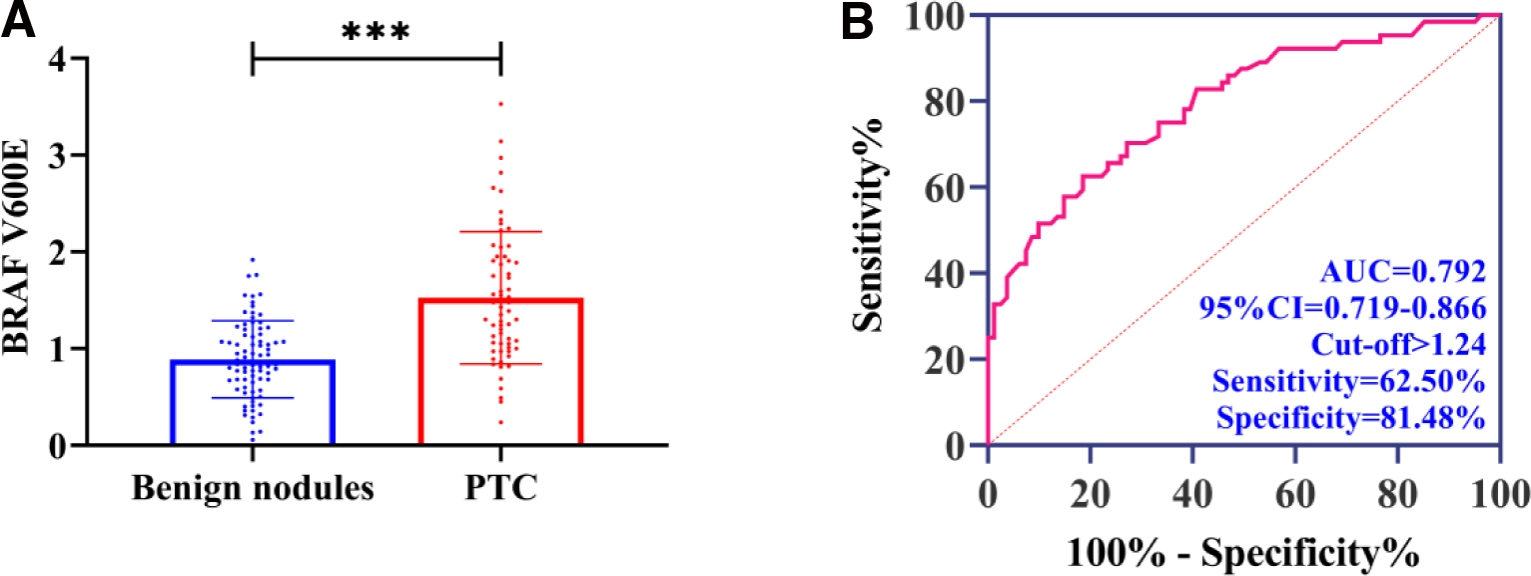
Diagnostic performance of serum BRAF V600E mutation for distinguishing papillary thyroid carcinoma from benign thyroid nodules. (A) Comparison of serum BRAF V600E between patients with papillary thyroid carcinoma (PTC) and benign thyroid nodules. Data are presented as mean ± standard deviation with individual data points. ***P < .001. (B) Receiver operating characteristic (ROC) curve for serum BRAF V600E in the diagnosis of PTC. The area under the curve (AUC) was 0.792 (95% CI: 0.719–0.866), with an optimal cutoff value of 1.24, yielding a sensitivity of 62.50% and specificity of 81.48%. BRAF = v-Raf murine sarcoma viral oncogene homolog B1, CI = confidence interval.

The assay demonstrated a sensitivity of 62.50% (95% CI: 49.5–74.4) and a specificity of 81.48% (95% CI: 71.2–89.2), with an overall accuracy of 72.41% (Table 3). ROC analysis yielded an AUC of 0.792 (95% CI: 0.719–0.866), with an optimal cutoff value of 1.24 (Fig. 1).

These findings support its role as a noninvasive diagnostic adjunct with comparable specificity to US-FNAC.

### 3.3. Diagnostic performance in poorly differentiated PTC

**Figure 2.**
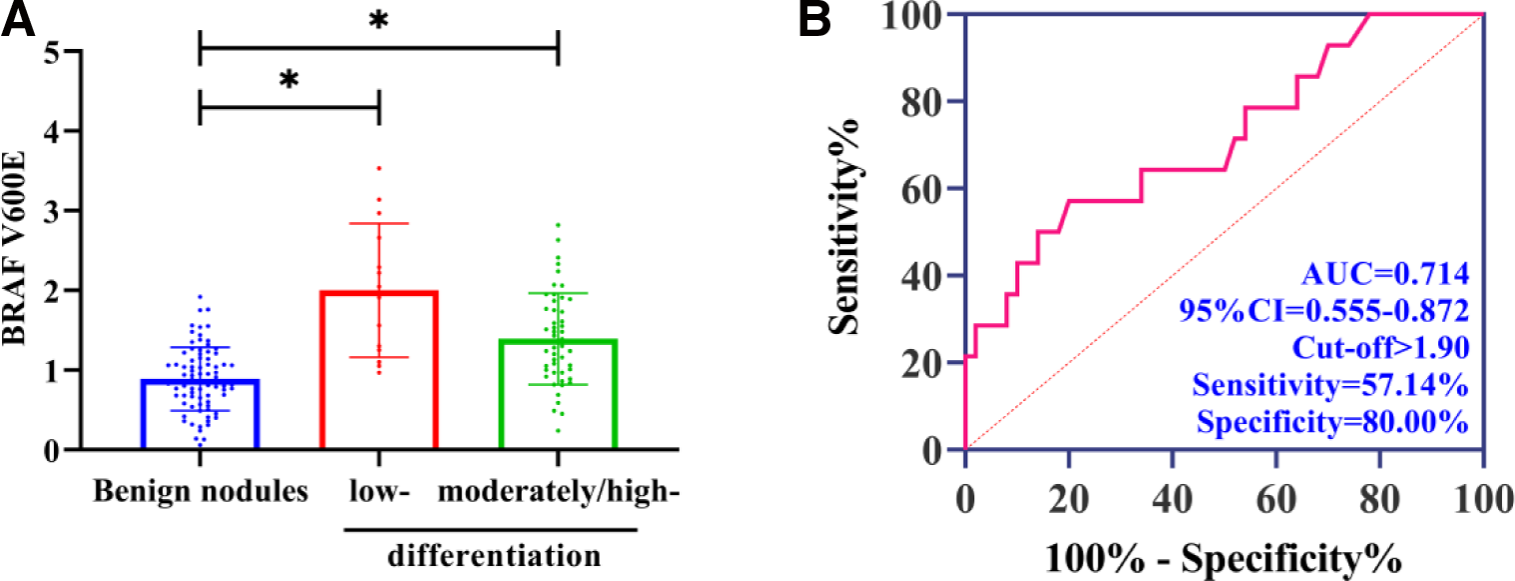
Diagnostic performance of serum BRAF V600E for differentiating poorly differentiated papillary thyroid carcinoma. (A) Comparison of serum BRAF V600E among benign nodules, poorly differentiated PTC, and moderately/highly differentiated PTC. Data are presented as mean ± standard deviation with individual data points. *P < .05. (B) ROC curve analysis of serum BRAF V600E for identifying poorly differentiated PTC. The AUC was 0.714 (95% CI: 0.555–0.872), with an optimal cutoff value of 1.90, corresponding to a sensitivity of 57.14% and specificity of 80.00%. AUC = area under the curve, BRAF = v-Raf murine sarcoma viral oncogene homolog B1, CI = confidence interval, PTC = papillary thyroid carcinoma, ROC = receiver operating characteristic.

In poorly differentiated PTC, the optimal cutoff value was 1.90. The assay achieved a sensitivity of 57.14% (95% CI: 28.9–82.3) and a specificity of 80.00% (95% CI: 68.2–88.9) (Fig. 2), with an AUC of 0.71 (Table 5).

**Table 5:** Diagnostic performance of serum BRAF V600E in subgroup analyses.

Subgroup	n	Cutoff	Sensitivity (%)	Specificity (%)	AUC (95% CI)
Poorly differentiated PTC	14	1.90	57.14 (28.9–82.3)	80.00 (68.2–88.9)	0.71 (0.58–0.84)
Micro-PTC (≤1 cm)	17	1.90	94.12 (71.3–99.9)	74.47 (63.0–83.9)	0.841 (0.78–0.97)

Values in parentheses represent 95% confidence intervals. Optimal cutoff values were determined using the Youden index. Subgroup classification was based on postoperative histopathological findings.

AUC = area under the receiver operating characteristic curve, CI = confidence interval, PTC = papillary thyroid carcinoma.

These results indicate limited sensitivity in this subgroup.

### 3.4. Diagnostic performance in papillary thyroid microcarcinoma (micro-PTC)

**Figure 3.**
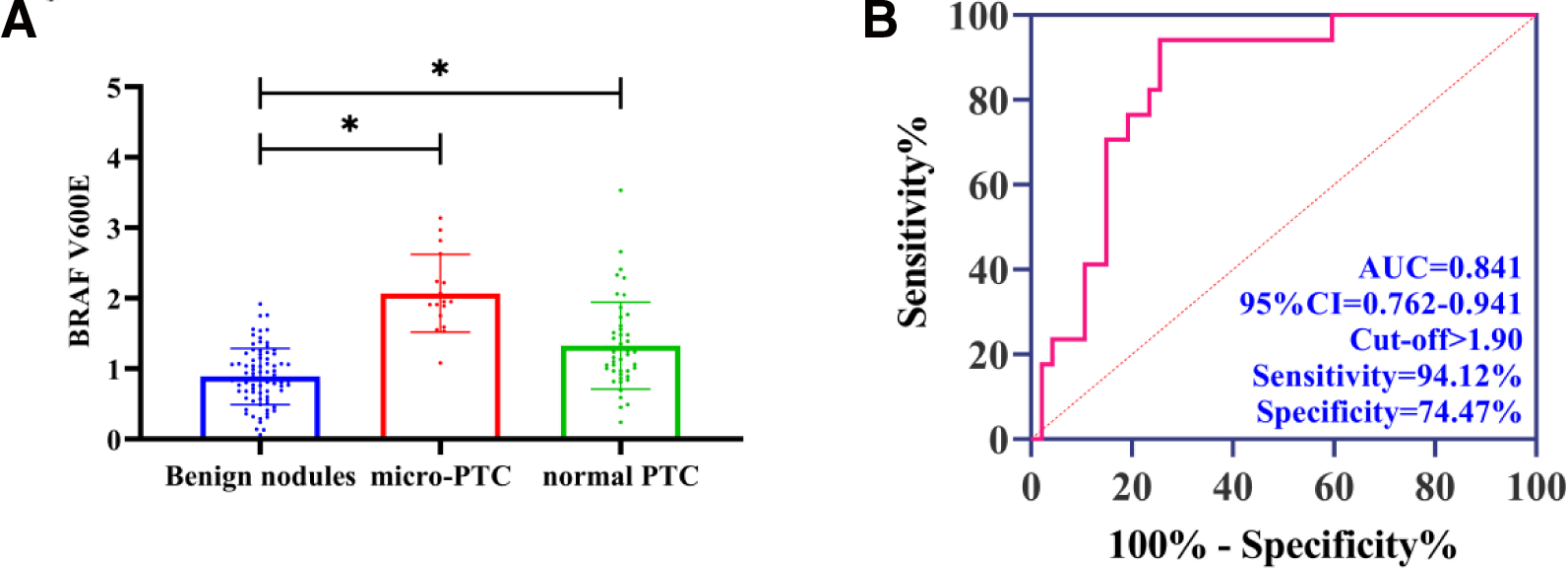
Diagnostic performance of serum BRAF V600E for detection of papillary thyroid microcarcinoma. (A) Comparison of serum BRAF V600E among benign nodules, papillary thyroid microcarcinoma (micro-PTC, ≤1 cm), and conventional PTC. Data are presented as mean ± standard deviation with individual data points. *P < .05. (B) ROC curve analysis of serum BRAF V600E for detecting micro-PTC. The AUC was 0.841 (95% CI: 0.762–0.941), with an optimal cutoff value of 1.90, yielding a sensitivity of 94.12% and specificity of 74.47%. AUC = area under the curve, BRAF = v-Raf murine sarcoma viral oncogene homolog B1, CI = confidence interval, PTC = papillary thyroid carcinoma, ROC = receiver operating characteristic.

In micro-PTC (≤1 cm), serum BRAF V600E demonstrated markedly improved diagnostic performance. Using a cutoff of 1.90, sensitivity reached 94.12% (95% CI: 71.3–99.9) and specificity was 74.47% (95% CI: 63.0–83.9) (Fig. 3), with an AUC of 0.841 (Table 5).

This represents substantially higher sensitivity compared with US-FNAC in this subgroup.

### 3.5. Combined diagnostic model (US-FNAC + BRAF V600E)

The combined model integrating US-FNAC and BRAF V600E significantly improved diagnostic performance, achieving a sensitivity of 81.25% and specificity of 85.19%, with an overall accuracy of 83.45% (Table 6).

**Table 6:** Comparative diagnostic performance of US-FNAC, serum BRAF V600E, and combined model.

Parameter	US-FNAC	BRAF V600E	Combined model
Sensitivity (%)	68.75	62.50	81.25
Specificity (%)	83.95	81.48	85.19
PPV (%)	77.19	73.91	81.48
NPV (%)	77.27	71.11	85.00
Accuracy (%)	77.24	72.41	83.45
AUC (95% CI)	–	0.792 (0.715–0.869)	0.873 (0.812–0.934)
Youden index	0.527	0.439	0.664
LR+	4.28	3.38	5.48
LR−	0.37	0.46	0.22
DOR	11.57	7.35	24.91

Values in parentheses represent 95% confidence intervals. The combined model was constructed using multivariable logistic regression incorporating US-FNAC and serum BRAF V600E status. Optimal cutoff values were determined using the Youden index. Diagnostic performance was evaluated against histopathological findings.

AUC = area under the receiver operating characteristic curve, BRAF = v-Raf murine sarcoma viral oncogene homolog B1, CI = confidence interval, DOR = diagnostic odds ratio, LR− = negative likelihood ratio, LR+ = positive likelihood ratio, NPV = negative predictive value, PPV = positive predictive value, PTC = papillary thyroid carcinoma, US-FNAC = ultrasound-guided fine-needle aspiration cytology.

The AUC increased to 0.873 (95% CI: 0.812–0.934), which was significantly higher than either US-FNAC or BRAF V600E alone (DeLong test, *P* < .05; Table 7).

**Table 7:** Comparison of AUCs using DeLong test.

Comparison	ΔAUC	*P*-value
Combined vs US-FNAC	+0.081	.021
Combined vs BRAF	+0.081	.018

Differences in AUC between models were compared using the DeLong test. A two-sided *P*-value < .05 was considered statistically significant.

AUC = area under the receiver operating characteristic curve, BRAF = v-Raf murine sarcoma viral oncogene homolog B1, US-FNAC = ultrasound-guided fine-needle aspiration cytology.

These findings indicate that the combined model improves both sensitivity and rule-out capability, as reflected by the reduced false-negative rate compared with US-FNAC alone.

## 4. Discussion

In this prospective diagnostic study, we demonstrated that serum-based detection of the BRAF V600E mutation provides reliable diagnostic performance for PTC, with particular strength in clinically challenging scenarios. Although the overall sensitivity of BRAF V600E detection (62.50%) was slightly lower than that of US-FNAC (68.75%), its comparable specificity and favorable AUC (0.792) support its role as a noninvasive complementary diagnostic tool. Importantly, the combined diagnostic model integrating US-FNAC and BRAF V600E significantly improved overall performance, achieving an AUC of 0.873 with statistically significant superiority over either modality alone, highlighting the added clinical value of multimodal diagnostic strategies.

US-FNAC remains the cornerstone for preoperative evaluation of thyroid nodules; however, its diagnostic accuracy is inherently dependent on sampling adequacy, lesion characteristics, and operator expertise.^[11]^ In the present study, the sensitivity of US-FNAC (68.75%) was consistent with previously reported ranges of 60 to 75%,^[12]^ whereas its specificity (83.95%) was slightly lower than that reported in some studies (90–95%).^[13]^ This discrepancy may be explained by the inclusion of a higher proportion of low-risk nodules (TI-RADS 3), which can increase false-positive interpretations due to overlapping cytological features. These findings reinforce the known limitations of cytology, particularly in heterogeneous or small lesions.

In contrast, the BRAF V600E mutation signal offers a molecular-level diagnostic approach by directly targeting a key oncogenic driver in the mitogen-activated protein kinase signaling pathway. The mutation is highly prevalent in classical PTC and is associated with tumor aggressiveness, invasiveness, and disease progression.^[7,14]^ In our study, a significantly elevated Ct-derived BRAF V600E mutation signal was observed in PTC compared with benign nodules, consistent with its biological role in tumor initiation and progression. Although the overall sensitivity of BRAF testing was moderate, its specificity remained comparable to that of US-FNAC, supporting its utility as an adjunct rather than a replacement for cytology.

A key finding of this study is the markedly improved diagnostic performance of BRAF V600E in papillary thyroid microcarcinoma (micro-PTC). The sensitivity reached 94.12%, substantially exceeding that of US-FNAC (64.71%), with an AUC of 0.841. This observation is clinically significant, as micro-PTC often presents diagnostic challenges due to limited cellular yield and subtle cytological atypia, frequently resulting in indeterminate or false-negative FNAC results.^[15]^ From a biological perspective, BRAF V600E is considered an early driver mutation that occurs in the initial stages of tumorigenesis.^[16]^ Therefore, even in very small lesions, the presence of circulating tumor-derived DNA enables detection through sensitive molecular assays. These findings underscore the potential of serum-based molecular testing as a valuable tool for early detection and risk stratification in small thyroid nodules.

In contrast, the diagnostic performance of BRAF V600E for poorly differentiated PTC was relatively limited, with a sensitivity of 57.14% and an AUC of 0.71. This may reflect the increased genetic heterogeneity of poorly differentiated tumors, which often harbor additional or alternative molecular alterations beyond BRAF mutations. Nonetheless, the moderate specificity observed in this subgroup suggests that BRAF V600E may still contribute to diagnostic refinement when used in combination with other modalities. Indeed, previous studies have shown that BRAF mutations are associated with enhanced proliferative signaling, angiogenesis, and tumor aggressiveness, which may partially explain their persistence in certain high-risk subtypes.^[17]^

Compared with existing literature, which has primarily focused on the role of BRAF V600E testing in cytologically indeterminate nodules (Bethesda III/IV),^[18,19]^ the present study expands its application to a broader clinical context, including early-stage and low-risk populations. Our findings support the concept that molecular testing should not be limited to selected high-risk cases but may have broader applicability across different diagnostic scenarios. This aligns with the “decentralized deployment” strategy proposed by Sharma et al,^[20]^ advocating for more flexible integration of molecular diagnostics into routine clinical workflows.

### 4.1. Clinical implications

From a clinical perspective, serum-based BRAF V600E detection may serve as a complementary tool within the existing diagnostic pathway for thyroid nodules. In particular, it may provide added value in patients with small nodules or indeterminate cytology (Bethesda III/IV), where conventional US-FNAC may yield inconclusive results. The high sensitivity observed in micro-PTC suggests that serum-based molecular testing could aid in early detection and risk stratification, potentially reducing the need for repeat biopsies or unnecessary surgical interventions. However, given its moderate sensitivity in the overall cohort, serum BRAF V600E testing should be considered an adjunct rather than a standalone diagnostic modality.

### 4.2. Limitations

Despite these promising findings, several limitations should be acknowledged. First, this was a single-center study with a relatively limited sample size (n = 145), which may introduce selection bias and restrict generalizability. In addition, regional factors such as iodine intake and genetic background may influence BRAF V600E mutation prevalence and diagnostic performance. Second, the absence of longitudinal follow-up precluded evaluation of the prognostic value of serum BRAF V600E detection, including its association with recurrence and long-term outcomes. Third, histological subtypes of PTC (e.g., classic, follicular variant, and tall cell variant) were not analyzed separately, which may have obscured subtype-specific differences.

Furthermore, although several clinical variables were assessed as potential confounders, none significantly influenced model estimates; however, residual confounding cannot be excluded. Subgroup analyses, particularly for micro-PTC (n = 17) and poorly differentiated PTC (n = 14), were based on small sample sizes, limiting statistical power and the stability of diagnostic estimates. In addition, the combined diagnostic model was not subjected to internal or external validation, which may affect the assessment of its robustness.

Therefore, future studies should include larger, multicenter cohorts with diverse populations, incorporate appropriate validation strategies, and include longitudinal follow-up to confirm the generalizability and clinical utility of serum-based molecular diagnostics.

## 5. Conclusion

In this prospective study, serum-based detection of the BRAF V600E mutation demonstrated reliable diagnostic performance for PTC and provided complementary value to conventional cytology. While its overall accuracy was comparable to US-FNAC, BRAF V600E showed markedly enhanced sensitivity in detecting papillary thyroid microcarcinoma, highlighting its potential role in early-stage disease. Importantly, the integration of BRAF V600E with US-FNAC significantly improved diagnostic accuracy, supporting a combined diagnostic approach.

These findings suggest that minimally invasive molecular testing can enhance current diagnostic pathways, particularly in cases with small nodules or limited cytological yield. Further multicenter studies with larger cohorts and longitudinal follow-up are warranted to validate these results and to establish the clinical utility of serum-based molecular diagnostics in routine thyroid cancer management.

## Author contributions

**Conceptualization:** Longzhu Zhao, Qi Li.

**Data acquisition:** Jing Jiang, Xiuqing Wang.

**Data analysis:** Xiuqing Wang, Jia Xu, Qi Zhang.

**Data interpretation:** Xiuqing Wang, Jia Xu, Qi Zhang.

**Investigation:** Qi Li, Yiyan Lu, Xiangli Li, Jia Xu, Qi Zhang, Longzhu Zhao.

**Methodology:** Qi Li, Yiyan Lu, Xiangli Li, Jing Jiang, Longzhu Zhao.

**Writing – review & editing:** Qi Li, Longzhu Zhao.
